# Altered Lipid Metabolism in CNS Demyelination and Remyelination Are Key Elements Driving Progressive MS

**DOI:** 10.3390/ijms26178314

**Published:** 2025-08-27

**Authors:** Agata Matejuk, Szymon Matejuk, Halina Offner, Arthur A. Vandenbark

**Affiliations:** 1 Department of Immunology, Collegium Medicum, University of Zielona Góra, 65-417 Zielona Góra, Poland; 2 Department of Internal Medicine, University of South Alabama, 2451 University Hospital Dr., Mobile, AL 36617, USA; smatejuk@health.southalabama.edu; 3 Neuroimmunology Research, R&D-31, VA Portland Health Care System, 3710 SW U.S. Veterans Hospital Rd., Portland, OR 97239, USA; offnerva@ohsu.edu; 4 Department of Neurology, Oregon Health & Science University, 3181 SW Sam Jackson Park Rd., Portland, OR 97239, USA; 5 Department of Anesthesiology and Perioperative Medicine, Oregon Health & Science University, 3181 SW Sam Jackson Park Rd., Portland, OR 97239, USA; 6 Department of Molecular Microbiology & Immunology, Oregon Health & Science University, 3181 SW Sam Jackson Park Rd., Portland, OR 97239, USA

**Keywords:** lipids, cholesterol, demyelination, remyelination, MS

## Abstract

Lipids, together with water and proteins, constitute the essential structure of cell membranes, and in the CNS, critically contribute to the production, function, and maintenance of the myelin sheath. Myelin produced by oligodendrocytes (OLs) acts as an electric insulator and assures proper conduction of information. Three major fractions of myelin lipids are cholesterol, phospholipids, and glycolipids. These lipids not only sculpt the myelin landscape as a structural support for proteins, but they also play a crucial role in molecular interactions underlying processes of protein trafficking and signal transductions. The high lipid content of myelin makes it susceptible to lipid metabolism disorders. Disorders in systemic and local lipid metabolism may lead to loss of myelin integrity and stability, and potentially to CNS demyelination seen in neurodegenerative diseases, notably progressive multiple sclerosis, for which there are few effective therapies. Precise interactions among disorders in lipid metabolism, function of oligodendrocytes, and demyelination/remyelination events, including de novo myelin formation and myelin remodeling processes, may lay the foundation for novel therapeutics for progressive MS and other demyelinating CNS conditions.

## 1. Background

### 1.1. Protection and Repair of CNS Myelin by Finely Tuned Lipid Metabolism May Be Crucial for Prevention and Treatment of Multiple Sclerosis

Multiple sclerosis (MS) is a neurodegenerative, autoimmune disorder of the central nervous system (CNS) leading to progressive physical disability and neurological symptoms including cognitive impairment. MS has been categorized clinically into three discernable clinical phases—relapsing–remitting (RRMS), primary progressive (PPMS), and secondary progressive (SPMS)—which may be preceded by bouts of clinically isolated syndromes (CISs) including optic neuritis [1]. The diagnosis of MS is based on MRI examination and presence of IgG oligoclonal bands in cerebrospinal fluid (CSF), and more recently described markers including the central vein sigh, paramagnetic rim lesions, and kappa free light chains, as noted in the 2024 McDonald criteria. The selective perivenous and confluent primary demyelination with the destruction and loss of oligodendrocytes distinguishes MS from other diseases with focal white and gray matter lesions [2]. Although immune cell infiltration is a core aspect of MS pathology, more recent data indicate that early preclinical pathological changes, including oxidative stress, mitochondrial abnormalities, and disruptions in metabolic homeostasis, may precede overt immune activation and thus contribute to the initiation and/or advancement of the disease. Activation of immune cells causing the myelin damage in MS is driven by various proteins, but may also be triggered by cell membrane lipids such as cholesterol [3]. New lesions normally remyelinate as a result of differentiation and recruitment of oligodendrocyte progenitor cells unless interrupted by recurrent activity [4]. Some observations in MS and its animal model—EAE (experimental autoimmune encephalomyelitis)—show that demyelination and neurodegeneration may be triggered by soluble factors produced by lymphocytes and not necessarily by direct interaction between those cells and target tissue [2]. Vidaure et al. identified ceramide, a lipid factor, as a potential cytotoxic factor for neurons in the MS CSF [5]. In this study, the exposure to MS CSF induced oxidative stress and decreased expression of neuroprotective genes, while increasing expression of genes involved in lipid signaling and in the response to oxidative stress. Furthermore, Fischer et al. showed that neurons, myelin, and oligodendrocyte degeneration in active MS lesions resulted from intense lipid oxidation [6]. Such oxidative injury and mitochondrial damage are known to be key events leading to demyelination and neurodegeneration in MS pathology. The precise nature of soluble factors and/or those derived from lipid metabolic dysfunction resulting in MS demyelination remain largely undefined. However, it is known that immune regulation is highly dependent on metabolic processes, especially lipid metabolism. Moreover, the proper differentiation, survival, and functions of T lymphocytes, especially the maintenance of immune tolerance, is dependent on lipid turnover [7].

### 1.2. What to Know About Lipids

Lipids come in various forms. The simplest forms are free fatty acids (FFAs) that in serum and interstitial fluid are carried by albumins [8]. Fatty acids (FAs) can be esterified to triglycerides (TGs) that transport FA in association with chylomicrons, very-low-density lipoproteins, or phospholipids, which are the main components of cell membranes. Fatty acids are simple in structure and are built of a varied-in-length alpha chain with a carboxyl group. They can be divided into saturated, monounsaturated, and polyunsaturated forms. In general, saturated FAs are cytotoxic in contrast to monounsaturated, which are cytoprotective [7]. The unsaturated FAs, which carry a double bond, can occur in two isoforms, cis and trans. Some FAs are called essential, meaning that they are not produced internally but must be derived from diet. Short chain FA (SCFA) (2–6 carbon atoms) such as acetate, propionate, and butyrate are produced by gut lumen microbiota from fiber and dietary carbohydrate [9]. A growing body of research indicates that SCFAs, especially butyrate and propionate, can impact neuroinflammatory processes and demyelination by modulating microglial activity and T cell responses. For instance, SCFA administration in EAE models has been shown to reduce demyelination and enhance the development of regulatory T cells, highlighting their potential neuroprotective effects mediated through the gut-brain axis [10]. Preclinical findings and a recent study by Duscha et al. showing immunomodulatory potential of SCFAs warrant clinical trials investigating the efficacy of SCFA supplementation in MS [11,12]. Fatty acids are not only a source of energy but also are precursors for more complex lipids like cholesterol, membrane phospholipids and biologically active compounds like eicosanoids and leukotrienes. They also are part of some hormones and signaling molecules. Synthesis of FA and cholesterol starts with acetyl-CoA, a product of carbohydrate, lipid and protein catabolism, and involve about 23 enzymes to produce cholesterol and 12 enzymatic steps to produce FA [13]. Due to the BBB impermeability to cholesterol during normal conditions, its metabolism, transport and storage is independent from the periphery [14,15]. However, one of its transporter and a metabolite, 24S-hydroxycholesterol (24S-OHC) can freely pass the BBB and effect immunity not only within the CNS but also in the periphery by acting on liver receptors (LXRs) [16]. Activation of LXRs has an anti-inflammatory effect by inhibiting proinflammatory genes as well as effects on the remyelination process [17,18].

### 1.3. Role of Lipids in CNS Myelin

In the CNS, the most lipid rich organ, the proper cholesterol and lipoprotein synthesis and homeostasis carried out by glial cells not only reassures energy expenditure and proper myelination, but also the control of synaptogenesis [19]. Lipids, including cholesterol, phospholipids, glycosphingolipids with FAs as essential building blocks in myelin, constitute 80% of its dry weight [14,20]. Myelin, with a highly organized and compacted multi-membrane structure, is generated by Schwann cells (SCs) in the peripheral nervous system and by oligodendrocytes in the CNS. Besides this main function, these cells also provide metabolic as well as trophic support to axons [21]. Myelinization by oligodendrocytes critically depends on a constant synthesis of lipids, which also serve as an energy source in a low glucose environment [22]. Disrupted lipid metabolism greatly burdens the composition, stability and integrity of myelin. Defects in phospholipid, sphingolipids and long chain FA often occur in diseases with neurological presentation [23]. Structures containing cholesterol and oxidized or otherwise modified lipids may serve as potential immune targets in MS, particularly under conditions of oxidative stress and lipid peroxidation [3]. MS progression is now distinguished as having slower signal propagation due to energy deficits in axons, with a direct effect on lipid metabolism and an inability of OLs to rebuilt myelin. There is a potential interest in new therapeutic strategies based on lipid metabolism manipulation to boost myelin remodeling processes and remyelination of damaged neurons. Knowledge obtained from this constantly developing field of immunometabolism may lead to new therapeutics for a spectrum of demyelinating and neurodegenerative disorders, including MS. While disturbances in lipid metabolism are known to underlie inherited metabolic myelinopathies such as leukodystrophies, the focus of this review is on autoimmune-mediated myelinoclastic disorder, MS, in which lipid homeostasis is critically involved in preserving myelin structure and function.

In this article we will present new findings on the role of lipid metabolism in MS, with some reference to demyelination and remyelination.

## 2. The Role of Lipids in Immune Cell Function

Environmental metabolic substrate availability and cell-intrinsic programs determine immune cell fates. Immune regulation is highly dependent on metabolic processes, especially on lipid metabolism. It has been proven that lymphocyte development, differentiation, effector functions and survival rely on lipids [7]. For example, the maintenance of immune tolerance in general but particularly to antigens derived from food and bacterial flora by regulatory T cells resident in the mucosal layer of the colon require short chain FAs (SCFAs) such as acetate, propionate, and butyrate [9,24]. Activated T cells possess higher plasma membrane FAs and cholesterol levels [25,26]. Memory T cells preferentially generate energy from lipid oxidation rather than glycolysis and being quiescent, they are less dependent on organelle biogenesis. Both CD4+ and CD8+ subtypes possess adequate lipid receptors and sense quantity and quality of the intracellular and extracellular lipid content. Fatty acids are recognized by T cells via receptors such as CD36, G protein-coupled receptors (GPCRs), fatty acid-binding protein TM (FABP_TM_), and members of the fatty acid transport protein (FATP) family [7]. A variety of lipids including α-GalCer as the strongest activator, complexed with the CD1d molecule are directly recognized by natural killer T cells (NKTs) [27]. NKT cells constitute a heterogenous T cell population and are recognized as important immune regulators in MS [28]. Remains to be clarified why in MS patients invariant NKT cells are anergic to the endogenous myelin-derived lipids including α-GalCer [29]. Sulfatides, a major component of myelin glycolipids in in vitro and in vivo studies have been defined as negative regulators of T cell function and Th17 differentiation with the effect on demyelination [30].

Nuclear receptors and transcription factors that alter transcription of genes not only important for lipid metabolism but for T cell activation, proliferation, and differentiation into Th1, Th2, Th17, and Treg lineages belong to the PPARs (peroxisome proliferator-activated receptor) [31,32]. PPARs besides FAs preferentially bind to long-chain polyunsaturated fatty acids (PUFAs) [33]. The most ubiquitously distributed is PPARγ, with potent immunomodulatory function. In EAE, PPARγ deficiency selectively promoted Th17 differentiation, resulting in increased numbers of infiltrating Th17 into the CNS and higher disease severity scores [34]. Conversely, in vivo colitis models, PPARγ agonists were shown to attenuate inflammatory responses [35,36]. Similarly, the PPARγ ligand ciglitazone was found to enhance the induction of TGF-β-induced Treg (iTreg) [37], and PPARγ was found to inhibit Th responses by blocking transcriptional activity of the IL-2 promoter via NFAT [32,38,39]. Subsequently, a double-blind, randomized trial involving 227 patients with RRMS demonstrated that daily administration of the partial PPARγ agonist CHS-131 (also known as INT131) significantly reduced the incidence of new gadolinium-enhancing lesions over a 6-month period compared to placebo [40]. PPARα agonists, such as fenofibrate and gemfibrozil, have also shown promising preclinical effects in MS models by reducing neuroinflammation and promoting remyelination through immunomodulatory and metabolic pathways [41]. Although preclinical and some early clinical studies suggest potential benefit of PPARs, large-scale trials are needed to confirm their efficacy in MS therapy.

Transcription factors that participate in activation of all genes required for FA synthesis and cholesterol, two major elements of cell membranes, belong to sterol regulatory element-binding proteins (SREBPs) [13]. SREBPs are the molecular signals that underlie the metabolic reprogramming of CD8(+) T cells during the transition from quiescence to activation [42]. Recently, it has been shown that endothelial cells respond to inflammatory cytokine challenges by reducing the accessible cholesterol pool on the plasma membrane thereby inducing canonical SREBP processing and gene expression leading to inflammation [43].

Intrinsic and extrinsic cues determine the developmental fate of T cells. In the case of CD4 T cells, these include cytokines TGFβ, IL-6, IL-23, metabolic substrate availability, transcription factor expression [Foxp3, RAR-related orphan receptor gamma t (RORγt)], and the activity of key metabolic enzymes [7]. T effector cell have different metabolic requirement as compared to regulatory T cell subsets. The latter rely on FAs, whereas Teff rely on high glucose availability and higher levels of glycolysis [44] as observed for the glucose transporter Glut1 that is selectively essential for CD4 T cell activation and effector function [45]. Whether T cells develop into Th17 or Tregs depends on metabolic programming and enzyme pyruvate dehydrogenase kinase (PDHK1), a selective regulator of T cell differentiation and inflammation [46]. In EAE, inhibition of PDHK1 modulated immunity and protected animals from disease by down-regulating the number of Th17 cells and increasing Tregs. Th17 cells preferentially use acetyl-CoA to malonyl-CoA conversion in the cytosol (de novo FA synthesis) for production of membrane phospholipids, whereas Tregs preferentially take up exogenous FAs for this function [47]. By using single-cell RNA-seq in EAE mice, Kuchroo et al. identified CD5L/AIM as a general inhibitor of Th17 pathogenicity [48]. CD5L has been found to be highly expressed in ‘non-pathogenic’ Th17 cells as compared to pathogenic Th17 with the role of intracellular lipidome modulation by inhibiting FA synthesis and restricting cholesterol biosynthesis. Moreover, they showed that saturated fatty acid (SFA) increased whereas PUFA decreased binding of RORγt (transcription factor necessary for function of pathogenic Th17) to the *Il17* and *Il23r* loci. This study highlights the importance of lipid metabolism in balancing immune protection and Th17 cell-induced pathology (e.g., MS). In general, lymphocytes are sensitive to lipotoxicity by exposure to elevated FFAs that lead to cellular damage and ultimately cell death [49,50]. Foxp3 in Tregs reprograms T cell metabolism to function in low-glucose and high-lactate environments and drives FA oxidation (FAO) and oxidative phosphorylation (OXPHOS), reassuring protection from lipotoxicity [49,51].

Cholesterol and its metabolites, particularly oxysterols acting through transcription factors such as LXRs, SREBPs and the GPCR EBI2 have been proven to have crucial effects on the innate and adaptive immune responses, including proliferation of cells and their differentiation and migration [25]. Human CD4+ T cell function is controlled by LXR by direct regulation of glycosingolipid synthesis [52]. Cholesterol derivatives regulate the phagocytosis of macrophages, homing of B cells to lymph nodes, inflammasome activation, anti-tumor responses of CD8 T cells, neutrophil traps and control of viral replication [25,53,54,55,56,57]. LXRs (LXRα and LXRβ) are expressed mostly on adipose tissue, liver, lymphocytes and brain and respond to cholesterol precursors and oxysterols, including desmosterol, 24S-hydroxycholesterol, 25-hydroxycholesterol, and 27-hydroxycholesterol. They control genes involved in cholesterol and FA biosynthesis as well as suppress the activity of genes under control of NF-κB and AP-1 [58,59]. In the EAE model some oxysterols like 7α,25-dihydroxycholesterol have been reported to play pro-inflammatory roles via increased migration of pathogenic CD44+CD4+ cells to the CNS [60]. A positive role for oxysterols has been established for Th17 function since they may serve as potential endogenous RORγt ligands in promoting the differentiation of mouse and human CD4+ Th17 cells [61]. The first in vivo evidence about the crucial role of LXRs in cholesterol homeostasis in the brain was reported in LXRα and -β deficient mice that had increased infiltration of inflammatory cells in the spinal cord and more severe demyelination leading to degenerative processes in the brain [62]. In EAE, LXR regulated Th17 cell differentiation and profoundly ameliorated disease severity [63]. In another EAE study, it was shown that LXR agonists may suppress the development of EAE, at least in part, through suppression of effector cytokines by Th1 and Th17 cells [64]. A dual role for LXR in controlling inflammation, including suppression of pro-inflammatory T cells and reciprocal induction of regulatory T cells, was discovered through use of pharmacological LXR agonists [65]. This duality reflects the context-dependent roles of LXR signaling: while endogenous oxysterols may promote Th17 differentiation via RORγt activation, synthetic LXR agonists can suppress Th1/Th17-mediated inflammation and promote Treg induction, likely due to broader transcriptional reprogramming effects. New research shows that LXR-mediated lipid metabolism pathways found to be dysregulated in CD4+ T cells from patients with RRMS could be fixed by stimulation with LXR-agonist GW3965 able to normalize membrane cholesterol levels, and reduced proliferation and IL17 cytokine production [66]. Of note, LXR and PPAR agonists stimulated phagocytosis in TREM2+ (triggering receptor-expressing myeloid) cells [67]. TREM2 is a central hub in the innate immune response and has been found to be activated by various proteins and lipids [68]. Recently, Ferrara et al. reported that TREM2 is a positively regulated thyroid hormone target gene and that the CNS-penetrating thyromimetic agent, sobetirome, renders TREM2 “druggable” since it was able to increase expression and signaling through the TREM2 pathway [69]. Previously, the same group showed that sobetirome inhibited myelin and axonal degeneration and oligodendrocyte loss in EAE [70]. Lipidomic approach to the study of human CD4 T lymphocytes in MS showed altered phospholipids and increases in cardiolipins that might reflect mitochondrial misfunction [71]. Lipidome studies with new analytical techniques in general open new directions for future research on lipid biology, as is the case of microglia for deciphering lipid classes and metabolism in pathophysiology not only in Alzheimer disease (AD) but also other neurodegenerative disorders like MS [72].

## 3. Inflammation and Metabolic Dysfunction

**Figure 1:**
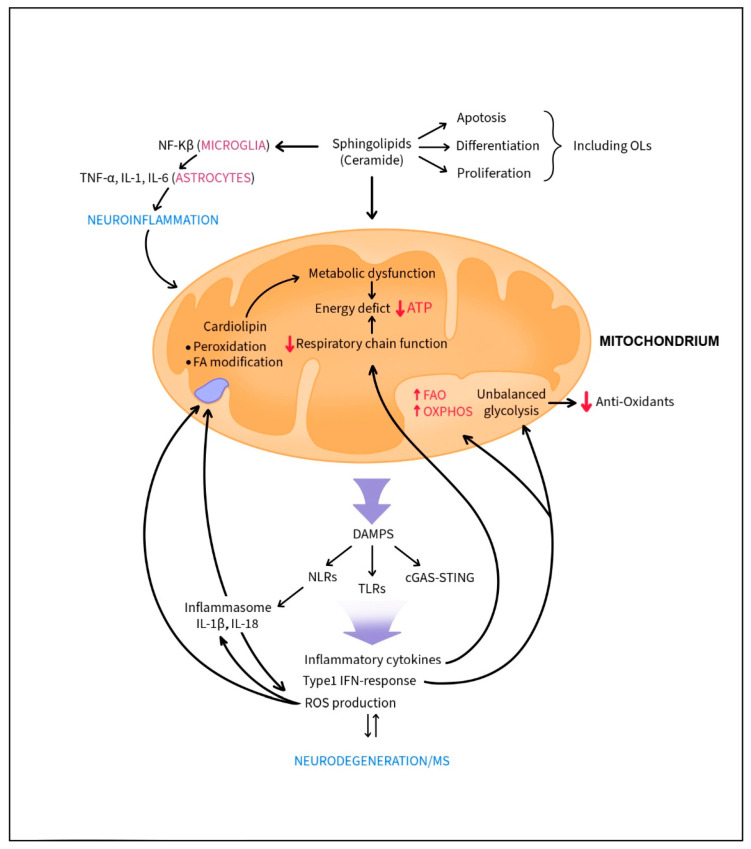
Dysfunction in metabolic and mitochondria dynamics boosts neuroinflammation and neurodegeneration observed in MS. Mitochondrial release of DAMPs triggers NOD-like receptors (NLRs), Toll-like receptors (TLRs) and cGAS-STING activation and promotes inflammatory cytokines, chemokines, and reactive oxygen species production. Proinflammatory cytokines cause mitochondrial imbalance. Type 1 IFN responses affect oligodendrocyte (OL) glycolysis and unbalanced glycolysis lowers the expression of anti-oxidants. Cardiolipin, an unsaturated phospholipid in the inner mitochondrial membrane, is especially vulnerable to reactive oxygen species (ROS). Its peroxidation and fatty acid arrangement and modifications are associated with metabolic dysfunction and energy crisis. Energy deficits further trigger the inflammatory responses and neurodegeneration. Upregulated ceramide levels (e.g., sphingolipids) activate NF-κB signal transduction, shifting the glial response towards inflammation and prompting the release of pro-inflammatory cytokines from astrocytes. This skewing of immune responses towards neuroinflammation can affect apoptosis, differentiation and proliferation of OLs.

There is a link between inflammation and metabolic imbalance and in the brain both phenomena have profound effects on demyelination and neurodegeneration. During CNS pathology resident glial cells like microglia and astrocytes are activated and able to promote or dampen the neuroinflammation [73,74,75,76]. Metabolic dynamics also plays a critical role in the development of MS [77,78]. Activation of microglia and astrocytes from ongoing inflammatory responses mandates high energy consumptions inducing metabolic alterations with direct effect on mitochondrial function. Mitochondrial fusion, fission and mitophagy form an essential axis of mitochondrial quality control, and mitochondrial architecture dynamics regulate bioenergetic efficiency and energy expenditure [79]. Besides the role of mitochondria as energy suppliers, they also play an important role in immune responses, whereas their dysfunction promotes neurodegeneration (Figure 1).

Mitochondrial DNA can trigger type I IFN responses, and alterations in mitochondrial dynamics may respectively induce either helpful or harmful immune reactions partially via secretion of cytokines such as IL-10 and TNF-a [80]. Dysfunction in metabolic and mitochondria dynamics boosts innate immune responses by mitochondrial release of DAMPs that trigger NOD-like receptors (NLRs), Toll-like receptors (TLRs) and cGAS-STING activation, promoting inflammatory cytokines, chemokines and reactive oxygen species production [81]. There is a strong correlation between mitochondrial DAMPS and type I IFN responses in accelerating neurodegenerative diseases. Autocrine action of IFN-I induces prominent shifts of the cellular metabolism, as augmented by FAO and OXPHOS [82,83]. Moreover, unbalanced glycolysis lowers the expression of anti-oxidants that facilitate oxidative damage. In MS, inflammatory cytokines like IFN-γ, IL-1 and TNF-α cause mitochondrial imbalance and that in OLs, glycolysis is highly impaired by IFN-I [84,85,86]. In astrocytes, IFN-γ and autophagy-related proteins upregulated by LPS promote mitochondrial autophagy (called mitophagy), a beneficial process that restores tubular mitochondrial networks after inflammatory responses [87]. It was also reported that PTEN—induced putative kinase 1 (PINK1), a mitophagy inducer, is crucial for microglial secretion of IL-10 and reduction of TNF-α levels via mTORC1 inhibition and consequently, decreased inflammation [88]. Cytokine activated astrocytes and microglia upregulate IL-1β and NFAT which not only regulates T cell activation, but also mediates astrocytic inflammatory responses. Specifically, NFAT signaling in astrocytes contributes to cytokine production and neurotoxicity [89]. Proinflammatory conditions adversely affect mitochondrial respiratory chain function with improper function of enzymes used in tricarboxylic acid cycle and oxidative phosphorylation leading to an energy deficit [90,91]. Energy deficit further triggers the inflammatory responses and neurodegeneration [92]. Demyelinated axons are more vulnerable to energy deficit than myelinated axons particularly in the presence of inflammation [93].

Mitochondrial DNA activation of the inflammasome and release of IL-1β/IL-18, potent cytokines that activate other proinflammatory cytokines, also boosts production of reactive oxygen species (ROS). Brain uses as high as 90% of tissue oxygen demand and is characterized by high oxygen metabolism for energy production in mitochondria and high production of ROS [94]. Lipids including polyunsaturated fatty acids abundantly present in CNS membranes and myelin are particularly vulnerable to high production of detrimental ROS. Interaction of ROS with NO creates peroxynitrite, a highly reactive agent. OPCs with low anti-oxidant capacity are especially endangered by oxidative stress that limits their abilities to mature and form myelin-forming oligodendrocytes (OLs) [92,95]. Reactive oxygen and nitrogen species induce NLRP3 and as a consequence, IL-1β secretion-boosting inflammatory responses. One of the lipids that is dangerously affected by ROS is cardiolipin, an unsaturated phospholipid in the inner mitochondrial membrane described in several different isoforms present in diverse regions and cells of the CNS [96,97]. Cardiolipin plays an important role in mitochondrial bioenergetic processes since it generates an electrochemical gradient that is used to produce ATP [98] (Figure 1). Abnormalities in cardiolipin (e.g., its peroxidation, fatty acid arrangement and modifications are associated with several pathophysiological situations, not only degenerative or age-related, but also associated with hypo–hyperthyroid states, heart ischemia/reperfusion, heart failure and diabetes [98]. Moreover, it has been suggested that cardiolipin may activate a subset of gamma-delta T cells (Tγδ) [99].

Sphingolipids, the main component of myelin, and their precursor, ceramide are potent lipid second messengers modulating diverse cellular signaling pathways (e.g., immune regulation that promotes apoptosis versus differentiation and proliferation versus growth arrest) in different cells, including OLs [77,100] (Figure 1). Ceramide has been shown to act directly on mitochondria and is able to induce the inflammasome [101,102]. Of note, ceramide’s induction of inflammasome senses obesity-associated danger signals and contributes to obesity-induced inflammation and insulin resistance [102]. An imbalance in ceramide metabolism can lead to various chronic diseases, including metabolic as well as neurodegenerative diseases [103]. Lipidomic studies have revealed a threefold increase in ceramide levels in the brains of AD patients compared to healthy controls [104]. In microglia, upregulated ceramide levels activate NF-κB signal transduction, shifting the glial response towards inflammation and prompting the release of TNF-α, IL-1β, and IL-6 from astrocytes. Of interest, dysregulated levels of ceramides and downstream metabolites have been found in white blood cells and plasma of MS patients [105].

In the CFS of patients with progressive MS, high levels of ceramides were reported with a positive correlation with neurological clinical scores [77,106]. Recently reported in the EAE model, neuronal-specific ablation of ceramide C16 synthetic enzymes protected from the mitochondrial dysfunction and severe form of the disease [107].

## 4. The Myelin Manufacture: Role of Oligodendrocytes and Lipids

Myelin abnormalities are seen not only in MS but also in neurodevelopment as well as other neurodegenerative disorders [108,109,110]. Widespread myelination occurs in the first few years of childhood up to middle age as a result of intensive expansion of the oligodendrocyte population derived from oligodendrocyte precursor cells (OPCs) also called NG2(+) cells. OPCs control homeostasis in adult brain by surveying their local environment and as needed are available to replace myelinating oligodendrocytes to participate in tissue repair. There is scarce remodeling of existing myelin since once formed, myelin is quite stable [111,112]. Reduction of the myelin sheath with age is accompanied by a decline in the oligodendrocyte population. During MS progression, the depletion in number and function of oligodendrocytes and OPCs is the major cause of disturbed remyelination [113,114]. Thus, although OPCs initially proliferate and attempt to restore myelination after injury, in progressive MS, both their numbers and remyelinating capacity decline, possibly due to chronic inflammation, aging, and inhibitory environmental signals.

**Figure 2:**
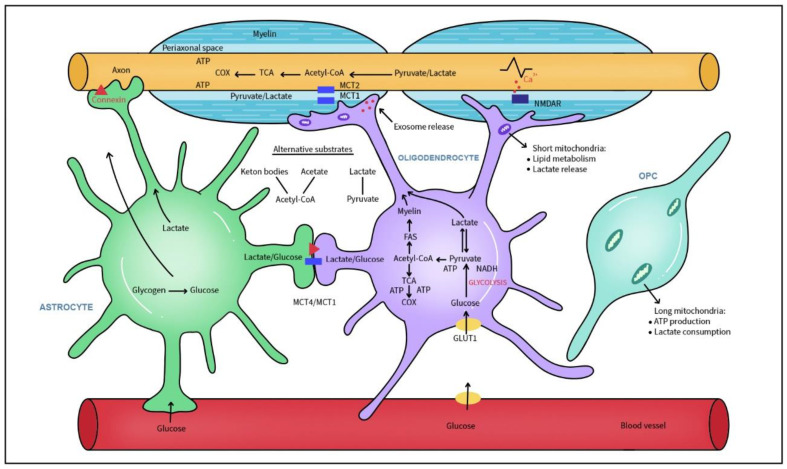
Metabolic interconnections between glia and axons. Myelinating oligodendrocytes (OLs) sense axonal activity through NMDA receptors and supply metabolites, energy substrates like glucose, lactate and pyruvate for axonal energy production via monocarboxylate transporters (MCTs) and exosomes. Additionally, astrocytes deliver energy sources through gradient-diffusion along MCTs and connexin channels. OLs and astrocytes are glycolytic and convert glucose from blood and glycogen (stored in astrocytes) to pyruvate and lactate. Lactate is converted to pyruvate which fuels mitochondrial ATP production. Alternative energy substrates are acetate and ketone bodies. Metabolites like acetyl-CoA are used for fatty acids (FAs) and cholesterol synthesis, both necessary for building up the myelin sheath. Short mitochondria in the myelin sheath and mature OLs favor lipid metabolism and lactate release in contrast to long mitochondria present in OPC.

A single OL can myelinate up to 50 axons, wrapping as many as 150 layers of myelin membrane around each axon [27]. Besides myelination, OLs play a critical role in delivering energy substances to axons of CNS neurons (Figure 2).

Saab et al. revealed a novel aspect of neuronal energy metabolism in which activity-dependent glutamate release enhanced oligodendroglial glucose uptake and glycolytic support of fast spiking axons [115]. Axonal energy demand and glutamate release trigger NMDA receptors in OLs with a direct effect on glucose transporter GLUT1 translocation to the membrane, glycolysis boost and lactated transport to axons. Dysfunction of myelinic NMDA receptors during chronic MS plaque formation dysregulates the energy supply between OLs, myelin and axons [115]. The axon-myelin unit not only mechanically supports signal transduction but is a functional unit where the dynamic communication between axons and their myelin-forming oligodendrocytes critically support bioenergetic axonal maintenance, the loss of which directly contributes to MS progression [116]. Both pyruvate and lactate are delivered to the periaxonal space via the monocarboxylate transporter 1 (MCT1) and neuronal isoform MCT2 for neuronal mitochondrial ATP production [117,118]. MCT1-null mice developed significant axonal degeneration with concomitant hypomyelination [119]. Glucose or pyruvate/lactate utilization is tightly regulated and is dependent on axonal energetic demands. Demyelination reduces the ability of OLs to generate lactate, resulting in reduced ATP production in axonal mitochondria and their dysfunction. Axonal ion dyshomeostasis weakens action potential propagation and activates enzymes such as phospholipases that further damage axonal structure. Glycolipids and phospholipids composed of FAs comprise the largest portion of myelin membrane lipids [120]. In contrast to other membranes, myelin possesses extraordinary stability associated with lipid composition containing high levels of saturated, long-chain fatty acids, 26% glycospingolipids and 30–40% cholesterol [121,122]. Moreover, myelin is rich in plasmalogens (etherlipids) with saturated long-chain fatty acids and α-hydroxylated galactosylceramide that are responsible for the tight packing of lipids in myelin [123,124].

Most OLs generate up to 100 membrane turns in a relatively short time and require high energy expenditure, with about 6.84 × 10^21^ ATP molecules to produce 1 g of myelin protein, 48-fold of that per 1 g of lipids [125]. This high energetic demand is provided by long, tubular mitochondria able to ensure effective, high rate OXPHOS that takes place before and during the myelination process [126]. Interestingly after myelination is complete, OLs rely less on mitochondrial respiration. In contrast to neurons, mature oligodendrocytes have low OXPHOS and higher glycolytic rate as supported by studies with knocked out OXPHOS in OLs [118]. Lack of OXPHOS in developing OLs led to severe demyelination in contrast with mature OLs which showed no effects on myelin or axonal function. Thus, it appears that mature OLs switch to a glycolytic state that provides the lactate to underlying axons via the myelin sheath. After myelination is complete, mature oligodendrocyte mitochondria are reduced in number and size. Mitochondria are also present within the myelin sheath, but their low density and small size indicate they are not important for OXPHOS, with the suggestion that those mitochondria are specialized for lipid metabolism and Ca^2+^ homeostasis [126]. While mature oligodendrocytes typically contain smaller and fewer mitochondria under physiological conditions, several studies have shown that mitochondrial biogenesis and size increase during progressive MS, likely as a compensatory response to energy failure and axonal damage [127,128]. Glucose is the main energy source during myelination process but OLs can also use lactate not only for ATP generation but as a precursor of lipids for carbon skeletons to synthesize myelin lipids [129,130]. Additionally, oligodendrocyte progenitor cells use lactate for differentiation and propagation [131]. Under low glucose availability, OLs change their metabolism from glucose to lipid metabolism based on FAs mitochondrial β-oxidation to create acetyl-CoA, a substrate for mitochondrial OXPHOS. In this scenario, lipid-rich myelin itself becomes a local energy reserve. FA oxidation that involves mitochondria and myelin-associated peroxisomes contributes to energy generation in OLs, but also to axonal ATP and conductivity. In a mouse model, reduced glucose availability caused a decline of GLUT1 and the gradual loss of myelin in a range of neurodegenerative diseases with underlying hypometabolism. In the starved *Drosophila* brain, neurons of the fly olfactory memory center import MCTs ketone bodies (such as acetoacetate and β-hydroxybutyrate provided by the cortex glia) that use their own lipid stores as an energy substrate to sustain aversive memory formation [132].

**Figure 3:**
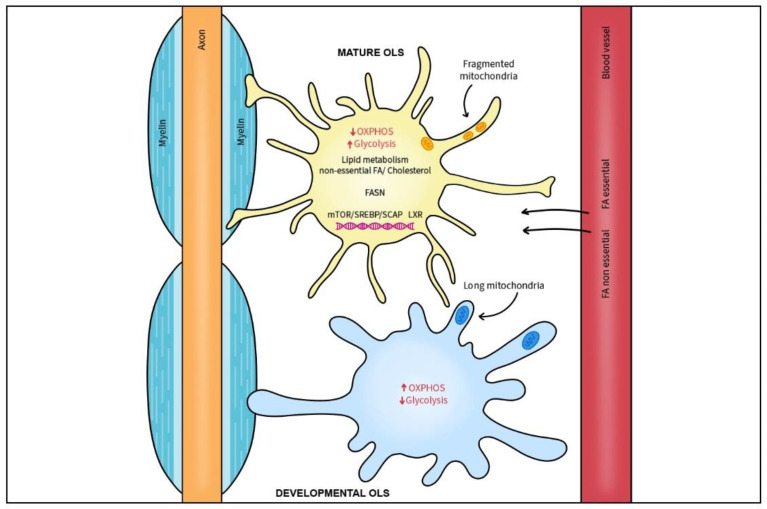
Myelination process relies on continuous lipid synthesis. The mTORC1/SREBP/SCAP axis as well as LXRs comprise key nuclear transcription factors in lipid and cholesterol homeostasis and are essential for endogenous lipid biosynthesis and myelination by OLs. Essential and non-essential FAs are derived from blood circulation or horizontal passage from local cells. FA synthase (FASN), an enzyme necessary for production of longer FAs is critical for timely onset of OL myelination and subsequent radial growth of CNS myelin. In contrast to developing OLs, mature OLs switch to a glycolytic state and have low OXPHOS. After myelination is complete, mature oligodendrocytes have smaller and fewer mitochondria that are specialized for lipid metabolism.

The myelination process relies on delivery of high quantities of lipids and is greatly dependent on continuous lipid synthesis [133,134]. With the exception of cholesterol that can only be produced within CNS, other lipid structures requiring fatty acids can be produced locally or delivered from the blood [120] (Figure 3).

Essential and non-essential FAs are derived from blood circulation or horizontal passage from local cells, and non-essential FA are endogenously produced by OLs and astrocytes [135]. In normal mouse brain development, formation of the myelin membrane requires lipid synthesis by oligodendrocytes as well as extracellular lipids provided by astrocytes and/or those delivered from the diet [135]. In general, cholesterol and FA synthesis relies on sterol regulatory element binding proteins (SREBPs) that are post-translationally activated by the sterol sensor SREBP cleavage-activating protein (SCAP). SREBPs have been found to be crucial for lipid synthesis in astrocytes [136,137] and the mTORC1/SREBP signaling axis is essential for endogenous lipid biosynthesis and myelination by OLs [138]. Depletion of mTOR or SCAP in OLs diminishes radial growth of myelin, thereby mimicking the effect of depleting squalene synthase, an essential SREBP downstream enzyme needed for cholesterol manufacture [135,139,140]. Additionally, OLs depleted of the SCAP and mTORC1 subunit, Raptor, have reduced levels of FA synthase (FASN), an enzyme necessary for production of longer FAs [135,138]. Indeed, depletion of FASN in OLs prevented the timely onset of myelination and subsequent radial growth of CNS myelin. However, FASN depletion did not play a major role in OL lineage progression based on normal expression of key analytical markers, Olig2, PDGFRα, and CC1 [133]. In that study, the lack of endogenous FA synthesis in OLs during development was partially compensated by increasing dietary lipid intake, implicating FA synthesis as crucial for efficient CNS remyelination.

Efficient remyelination based on activation, migration, proliferation and differentiation of CNS progenitors, including OPCs, is a critical process and the target of recent therapeutic approaches in diseases such as MS. Remyelination is dependent on de novo synthesis of FAs that sustain OPC-derived OLs [133,141] as well as Schwann cells, the myelinating cells of the peripheral nervous system (PNS) [142]. Besides FA that can be delivered from local production or blood, proper radial growth of myelin by OLs depends on cholesterol synthesis within CNS [120,140]. Cholesterol produced by glia complexed to apolipoprotein E-containing lipoproteins was found to be crucial for proper synapse development [19]. Within the CNS, cholesterol is synthesized from acetyl coenzyme A that requires energy and molecular oxygen and is produced locally because the BBB efficiently stops delivery of cholesterol from hepatic or dietary sources [143]. However, the shielding of the CNS of circulating lipids, including cholesterol, might not be so complete under conditions in which brain lipid metabolism is limited, since lipid synthesis in defective astrocytes could be improved by lipid supplementation [137]. Cholesterol is also a precursor for “neurosteroid” hormones, including estrogen and progesterone produced by glia and neurons that protect the brain from neurodegeneration and ischemic stroke [144,145]. The rate-limiting step in neurosteroid synthesis is the 18kDA translocator protein, TSPO, that transports cholesterol into mitochondria [146].

Key nuclear transcription factors called LXRs have a profound effect on myelination and remyelination processes via regulation of OLs function [147,148]. LXR deficient mice exhibited reduced myelin gene expression and thinner myelin sheaths. Conversely, activation of LXRs by either 25-hydroxycholesterol or synthetic TO901317 stimulated myelin gene expression leads to OL maturation and increased myelin production and remyelination processes [149]. More specifically, knockout of FASN (an LXR target gene) blocked FA synthesis, corrected myelin lipid composition and stabilized myelinated axons in a murine model [133]. Moreover, remyelination in aged mice was restored by stimulating reverse cholesterol transport via LXR target genes, ABCA1 and Apo-E [18].

Remyelination therapies aim to promote the direct repair of damaged areas in the CNS [150]. Drugs currently in use that successfully stimulate production of new myelin or repair of existing myelin include clemastine, benzotropine, quetiapine, ivermectin and GSK239512 [150,151,152,153]. The mode of action of such drugs includes direct effects on the differentiation, recruitment and survival of oligodendrocytes and interactions with H1 or H3 histamine or muscarinic acetylcholine receptors (e.g., M1). Recently, the striking effects of our DRhQ drug that inhibits CD74-dependent inflammation enhances myelin preservation and remyelination in EAE [154]. The studies to decipher the mechanisms of the beneficial effects of DRhQ on myelin regeneration are ongoing. Other drugs such as the humanized monoclonal antibody, Opicinumab, that targets the LINGO-1 glycoprotein (an inhibitor of axonal regeneration, myelination and neuronal survival) has been reported. However, although Renew and Renewed studies indicate improved electrophysiological results suggestive of remyelination after opicinumab treatment in patients with acute optic neuritis, no clear specific biomarkers of remyelination were detected in the Synergy study, a phase 2 trial investigating the safety and efficacy of this drug in MS patients [155,156]. Other remyelination therapies are based on replacing damaged cells by neural or mesenchymal stem cells that can differentiate into oligodendrocytes [157,158]. Cell-based therapies directly repopulate and substitute endogenous cell niches with OPCs or different stem cell types, creating the proper environment for reduced inflammation and reduction and modulation of inflammation [159,160]. Interestingly, the most recent study suggest that oligodendrocytes are present in MS lesions but are epigenetically silenced for myelin production [161]. Liu et al. identified a small-molecule epigenetic-silencing-inhibitor (ESI1) that promoted remyelination in animal models of demyelination by triggering nuclear condensate formation of SREBP1/2, a master lipid-metabolic regulator to promote lipid/cholesterol biosynthesis [161]. This study underscores the potential of targeting epigenetic reprogramming machinery to boost myelin production and regeneration.

## 5. Lipid Metabolism in Progressive MS

There is compelling evidence that the course of MS is closely related to changes in the metabolome [162,163,164,165]. Energy requirements are highest in neurons of the adult brain [166]. The lack of ATP; impaired calcium handling and increased ROS production due to mitochondrial defects are likely to play a major role in axonal dysfunction and degeneration in all forms of MS especially during progressive stages of MS [93]. Progressive MS is characterized by diffuse white and grey matter injury with smoldering lesions and mixed active/inactive chronic MS plaques [167], and is associated with over-activated microglia/astrocytes predominated by neurodegenerative processes where energy demand of demyelinated neurons cannot be compensated [168]. Increase of transmembrane potential, dysregulation of protein and fatty acid oxidation and peroxisomal dysfunction further deepen metabolic dysfunction [93,169]. Chronic metabolic dysfunction is linked to axonal pathology in MS; as shown by MRI measurement of N-acetylasparate; an amino acid synthesized in brain mitochondria [170]. N-acetylasparate synthesis is dependent on oxygen consumption and mitochondrial respiratory chain activity and has been found to be diminished in acute MS lesions reflected not only as an axonal loss but also as a metabolic signature of dysfunction [171,172]. NMR metabolomic studies of urine samples from MS patients revealed glycolysis and the synthesis and degradation of ketone bodies as potentially high-impact pathways [173]. Ketone bodies are associated with fatty acid metabolism and are used by brain as an alternative energy source [174]. Changes in FA, cholesterol, oxysterols and sphingolipids in general lipid metabolism well portray MS and other demyelination pathologies including ageing [18,175,176,177,178,179,180]. Faulty FA turnover such as an alteration in propionate metabolism caused a deficiency in FA metabolism that was linked to various neurological problems [173,181,182]. A noninvasive metabolite scan on the white matter of RRMS patients done by Vingara et al. showed decreased choline, a vital substrate for cell membrane’s two major phospholipids, phosphatidylcholine and sphingomyelin [183]. Analysis of untargeted high-resolution metabolomics of plasma samples to identify primary progressive MS-specific signatures showed alterations in glycerophospholipid and linoleic acid pathways [184]. Moreover; using higher field strength NMR spectroscopy (800 MHz), variations in energy and phospholipid metabolism in CSF samples from MS patients have been reported [185]. In another study, metabolic profiling of serum samples from MS patients were associated with disease severity and an altered pattern of sphingomyelin and lysophosphatidylethanolamine; a major phospholipid in cell membrane [186]. High concentrations of lactosylceramide that control the recruitment and activation of microglia and CNS-infiltrating monocytes and an enzyme involved in its synthesis, beta-1,4-galactosyltransferase 6 (B4GALT6), were detected in EAE and in MS CNS lesions [187]. Levels of ceramides (the structural backbone of shingolipids (SLs)) as products of de-novo synthesis and/or sphingomyelin degradation and their bioactive metabolites within sphingolipid (SL) metabolism pathways such as cytotoxic shingosine versus bioprotective Sph-1-phosphate (S1P) may determine OLs demise and demyelination process. Increased levels of ceramides such as C16:00; C18:00 and C20:00 and decreased levels of S1P have been found to cause OL’s apoptotic cell death in astrocytes in animal models of demyelination [188]. In 2010, Qin reported that neurons and OLs recycle S1P to ceramides and that increased levels of sphingosine and ceramide C16/18 caused cell death in cultured neurons and OLs as well as in cells from white matter and plaques in brains of MS patents [189]. In 2014, Vidaurre et al. showed that the CSF of patients with MS was enriched in C16/24 ceramides able to induce neuronal mitochondrial dysfunction and axonal damage [5]. Moreover, a more recent retrospective study in 2020 by Amatruda et al. found that plasma of subjects with progressive MS with a rapidly deteriorating disease course showed a significantly lower abundance of C18:2-LPA (lyso-phosphatidic acid-18:2) and increased levels of C20:0-HexCer (mono-hexosylceramide) [190]. Interestingly, in the same year (2020), Podbielska et al. employed targeted sphingolipidomics to elucidate the SL profiles in lesions in progressive stages of MS [191]. They found that chronic inactive lesions were characterized by decreased levels of dihydroceramide (dhCer), ceramide and sphingomyelin subspecies, whereas levels of hexosylceramide and Cer-1 phosphate (C1P) were significantly increased in comparison to normal CNS and active lesions. These exciting results prompted the authors to propose C1P as a potential new biomarker of the progressive stage of MS. Indeed, these results appeared to be an important advance based on supporting data from the previous studies cited above as well as another 2008 report from Wheeler et al. indicating that there was not an overall loss of lipid mass in white and grey matter in MS brains, but a reduction in sphingolipids and gain in phospholipid content during active disease [192]. A 2020 paper from Pousinis et al. provided additional concurrent data showing that metabolic pathway analysis of white matter from post-mortem cases of PPMS and SPMS revealed that the most altered lipid pathways between those two were glycerophospholipid metabolism, glycerophosphatidyl inositol anchor synthesis and linoleic acid metabolism [193]. The progressive phase of MS is predominated by neurodegenerative activity resulting in neuronal and axonal loss over and above the inflammatory demyelination. S1P appears to be a lipid mediator within the neurodegenerative sector of MS development. Elevated levels of S1P has been found in CSF and not in blood of MS patients and may reflect ongoing development of MS plaques in the CNS parenchyma [194]. Increased S1P has been associated with hyperactivation of astrocytes in the EAE model since lack of S1PR1 in astrocytes attenuated astrogliosis; a phenomenon associated with neurodegeneration in MS [195]. S1PRs expressed by immune and CNS cells are believed to be joints between neurodegeneration and S1P signaling. Modulation of S1PRs inhibited activation of murine and human astrocytes in vitro and disease progression in the animal model of SPMS [196]. Metabolomic studies done in EAE generally were in accordance with those obtained in MS patients; although dysregulations in the lipid pathways (e.g., linoleic acid) were detected in plasma of EAE mice [197]. Another study showed variations in long-chain phospholipids; and FAs accompanied by increases in oxidative stress and immune responses in the EAE model [198]. Poisson et al. found several pathways related to FA metabolisms with lower levels of ω-3 and ω-6 polyunsaturated FAs in EAE mice [199]. The treatment of resolvin D1; a downstream metabolite of ω-3; stopped the progression of EAE most likely by the polarization of monocytes/macrophages and microglia into the M2 phenotype and induction of regulatory T cells. Several studies showed a correlation between elevated levels of circulating low density lipoprotein cholesterol (LDL), total cholesterol, apolipoprotein B and oxidized LDL with adverse clinical and MRI outcomes in MS [200]. The data indicate that cholesterol and markers of cholesterol turnover have potential to be used clinically as biomarkers of disease activity [200,201]. In early MS, lipid profile variables, particularly total cholesterol and LDL levels were associated with inflammatory MRI activity measures [176,177]. In other studies, increased levels of total cholesterol, LDL, VLDL and Apo-B were associated with higher EDSS score [178,202,203]. However, recent meta-analysis examining the association between serum lipid profiles and cognitive performance in MS did not find significant correlations between HDL levels and cognitive outcomes [204]. Although a modest inverse relationship was observed between total cholesterol and MoCA scores, no consistent associations were found between lipid measures and other cognitive tests, suggesting that further well-designed longitudinal studies are needed to clarify these relationships.

An altered lipoprotein profile with dysfunctional HDL (abnormalities in HDL and LXRs or HDL oxidation to OX-HDL) was found in RRMS patients [205]. In contrast, higher levels of high-density lipoprotein cholesterol (HDL-C) in serum positively correlated with lower levels of BBB injury and decreased CD80+ and CD80+CD19+ cell extravasation into the CSF [206]. Increases in HDL-C and ApoA-I have protective associations with MRI measures of neurodegeneration in both RRMS and progressive MS over 5 years of follow-up [207]. HDL with its anti-inflammatory, antioxidant, and nitric oxide stimulus properties play a potentially critical role in MS [208]. HDL reduces leukocyte migration and BBB permeability by modulation of S1P, a main representative of sphingolipids and a crucial molecule in MS [209]. Increases in blood HDL levels have been observed in MS patients treated with fingolimod, an agonist drug that targets S1P receptors [210]. Of note, decreases in all cholesterol biomarkers following IFN-beta1a treatment have been associated with brain atrophy outcomes over 4 years [177]. LXRβ, that plays a crucial role in the regulation of cholesterol metabolism, was found to be upregulated in peripheral blood mononuclear cells in MS patients [211]. Progression of MS is directly linked to the neurodegenerative processes as an effect of mitochondrial dysfunction and abundant creation of ROS and formation of oxidized lipids [16]. Oxidative stress causes overproduction of oxysterols such as 7-KC, 7B-OHC and 24(S)-OHC, indicators of neurodegeneration in MS [16]. Ongoing neurodegeneration in MS can be predicted by patterns of oxysterol expression depending on the stage of disease [212,213]. Particularly, 24S-HOC, most abundant in the brain, can act locally, affecting the functioning of neurons, astrocytes, oligodendrocytes, and vascular cells reflecting brain cholesterol metabolism dysfunction [213]. In CNS cells, 24(S)-OHC effects cell proliferation and causes cell death, disrupting red-ox homeostasis [214]. Oxysterols and apolipoprotein changes were associated with conversion to SP-MS and disability progression in MS [215]. MS progression as a consequence of oxidative damage is strongly linked to high levels of ROS and lipid peroxidation products effecting neuronal and glial cells as well as BBB functionality [216,217].

Lipid lowering drugs like statins have been proposed for MS treatment. Statins possess neuroprotective effects by skewing differentiation of T cells toward Tregs [218,219,220]. Statins block HMG-CoA-reductase, an enzyme that generates mevalonate, a key intermediate in the cholesterol synthesis pathway. However, rather than lowering peripheral cholesterol levels, statins might inhibit isoprenoid intermediates of the mevalonate pathway linked to signalling pathways that regulate T cell autoimmunity [221,222]. One study demonstrated that simvastatin, with good CNS penetration, inhibited Th17 cell differentiation and lowered IL-17A, IL-17F, IL-21, and IL-22 secretion in in vitro-differentiated naive CD4(+) T cells from RRMS patients by blocking IFN regulatory factor 4 (IRF4) expression, which was identified as a key transcription factor for human Th17 cell differentiation [223]. The same group further reported that simvastatin inhibited IL-1beta, IL-23, TGF-beta, IL-21 and IL-12p70, and induced IL-27 secretion from DCs in RRMS patients, thus providing an inhibitory cytokine milieu for Th17 and Th1-cell differentiation [224]. Studies in the EAE model further demonstrated that Atorvastatin (Lipitor) promoted Th2 differentiation and suppressed APC involved in activation of antigen-specific T-cells [225]. However, although positive effects of statins in EAE did not show a proven therapeutic effect in RRMS [226], clinical trials for statins are ongoing in SPMS (ClinicalTrials.gov, number NCT00647348 and NCT03387670) [227]. A previous study showed that simvastatin monotherapy may have beneficial outcomes on disability levels in patients with SPMS [228]. Conversely, in RRMS, a systemic review and meta-analysis suggested a potential increase in disease activity [228]. At present the effects of statins on MS neuropathology seem controversial with conflicting outcomes [229]. Statins appear to be protective in the acute phase of MS through anti-inflammatory and antioxidant effects. However, statins lead to detrimental effects in the chronic phase of MS by reducing brain cholesterol and inhibiting the remyelination process [229]. Previously, it was shown that simvastatin inhibits remyelination after cuprizone-induced MS in mice [230]. A recent study shows anti-inflammatory and immunomodulatory effects of simvastatin in EAE, potentially alleviating symptoms of neurological dysfunction by regulating the balance between Th17 and Treg [231]. The detrimental and beneficial effects of statins could be dose dependent [229]. Thus, further clinical trials and prospective studies regarding the effect of statins on MS outcomes are encouraged. Among disease modifying therapies (DMTs) currently used in MS are modulators of S1PR like fingolimod, ponesimod, ozanimod, siponimod and amiselimod [190,232,233,234]. The only agent currently approved for SPMS treatment is Siponimod. It is a selective modulator of S1PR1 and S1PR5 which, by stabilizing BBB, mitigates the recruitment of peripheral immune cells to the CNS, and within the CNS modulates activation status of glia, and amelioration of neuronal and oligodendrocytes injury [235]. Several therapeutic trails with drugs such as dimethylfumarate (DMF) and biotin exhibiting anti-oxidative activity and focusing on normalization of energetic processes and regulation of lipid biosynthesis and metabolism have been reported, none with sufficient evidence to suggest a beneficial effect on disease progression [236,237]. Although agents such as S1P and DMF are not classical lipid-targeting therapies, they have garnered clinical interest due to their capacity to intersect with sphingolipid metabolic pathways, thereby modulating lipid-mediated signaling cascades and redox-sensitive lipid networks. Additional studies in animal models have consistently shown some therapeutic potential for drugs modulating sphingolipid metabolism such as Miglustat, oxysterols acting as LXR agonists or resolvin D1 (the latter works on PUFA metabolism) but it remains to be seen if these approaches will have a significant effect in suppressing chronic progressive MS [16,199,238,239].

## 6. Conclusions

Manipulating metabolic processes for therapeutic purposes has gained tremendous interest in recent years. Remyelinating strategies to assure regeneration and neuroprotection are favored in designing new therapies for progressive MS. As presented in this review, immune responses, myelination and remyelination processes are closely linked with lipid metabolic pathways, regulating energy intake and anti-inflammatory responses. Lipid signatures identified by lipidomic approaches may lead to the discovery of new biomarkers such as C1P that may be useful in prediction or monitoring the course of progressive MS, particularly important for patients with rapidly progressing disease. Pharmacological interventions affecting lipid homeostasis may be clinically favourable. Statins, drugs that inhibit cholesterol synthesis are one of the most prescribed drugs in the world. Statins are able to reduce endogenous RORγt ligand and as a consequence, Th17-mediated inflammatory conditions like MS and might be exploited for treatment strategies in autoimmune disorders. Similarly, drugs like gemfibrozil, and fenofibrate, the PPARα agonists prescribed for the treatment of hypertriglyceridemia, are able to promote Th2 responses and hold promise in treatment of autoimmune diseases. Identification of cross-talk among metabolic processes, lipid metabolism, immune regulation and myelination processes represent an exciting new area of autoimmune regulation.

## Data Availability

No new data were created.
